# Editors’ Note

**DOI:** 10.5195/ijt.2017.6221

**Published:** 2017-06-29

**Authors:** ELLEN R. COHN, JANA CASON

The current issue of the International Journal of Telerehabilitation (IJT) contains original work that explores the feasibility of telerehabilitation for a diversity of purposes: a health and wellness program for caregivers; a phonological awareness program for children with hearing loss; telepractice in a school setting for at-risk youth; occupational therapy home visits employing mHealth to facilitate discharge from the acute admission back to the community; speech and language intervention for primary school-aged children; and a commentary on the status of telerehabilitation in Pakistan. The issue’s lead article, “Privacy and Security in Multi-User Health Kiosks,” offers an audit-based protocol that can be used to assess whether a multi-user health kiosk is meeting privacy and security regulations such as HIPAA and HITECH.

A note about the high value that the IJT places on diversity, and how the editors actualize those values. The IJT encourages contributions from international contributors and honors dialectal differences. There are also differences in the terminology used between and within professions. In this issue, different authors variously refer to: telerehabilitation, telepractice, telehealth, and mHealth. Again, we respect such differences. The choice of terms is often particular to a profession’s preferred, or even mandated usage. These differences sometimes require negotiation when authors from different professions collaborate on a scholarly work.

Word usage that the IJT editors do attempt to discourage is use of the phrase “face-to-face” to denote communication that occurs within the same physical site. This is because videoconferencing enables face-to-face interactions to occur through video. The term “in-person” is therefore preferred to describe interactions within the same site. The use of the terms in-person to describe a ‘traditional’ therapy model and ‘face-to face’ to describe a telehealth model using videoconferencing seems to be gaining traction across the literature.

## CALL FOR SUBMISSIONS

The next volume of the International Journal of Telerehabilitation will be published in Fall, 2017. We cordially invite your submissions by September 1, 2017, and accept original research, case studies, viewpoints, technology reviews, book reviews, and country reports that detail the current status of telerehabilitation.

Our peer reviewers constitute a multi-disciplinary group, and include researchers and clinicians from each of the major rehabilitation disciplines, rehabilitation engineers, health information managers, information technologists, and others. We welcome new peer-reviewers and invite guest editors with ideas for special, thematically focused issues. The IJT publication team is agile and can add additional issues as warranted to ensure currency. Please contact Editor Ellen Cohn, PhD if you are interested: ecohn@pitt.edu.

Sincerely,

Ellen R. Cohn, PhD, CCC-SLP, ASHA-F,

IJT Editor

Jana Cason, DHS, OTR/L, FAOTA,

Senior Associate Editor

